# Risk factors for colonization and infection by *Pseudomonas aeruginosa* in patients hospitalized in intensive care units in France

**DOI:** 10.1371/journal.pone.0193300

**Published:** 2018-03-09

**Authors:** S. Hoang, A. Georget, J. Asselineau, A-G. Venier, C. Leroyer, A. M. Rogues, R. Thiébaut

**Affiliations:** 1 Inserm, Bordeaux Population Health Research Center, UMR 1219, Inria SISTM, Univ. Bordeaux, ISPED, Bordeaux, France; 2 Centre Hospitalier Universitaire Sud Réunion, Île de la Réunion, France; 3 Centre Hospitalier Universitaire de Bordeaux, Pole de santé publique, Service d’information médicale, Unité de Soutien Méthodologique à la Recherche Clinique et Epidémiologique, Bordeaux, France; 4 Service d’Hygiène Hospitalière Groupe Hospitalier Pellegrin, Bordeaux, France; 5 Centre de coordination des Comités de Lutte contre les Infections Nosocomiales Sud-Ouest, CHU Pellegrin, Bordeaux, France; National Yang-Ming University, TAIWAN

## Abstract

*Pseudomonas aeruginosa* (*P*.*aeruginosa*) remains a prominent nosocomial pathogen responsible for high morbi-mortality in intensive care units (ICUs). *P*.*aeruginosa* transmission is known to be partly endogenous and exogenous. Main factors have been highlighted but the precise role of environment in regard to antibiotics use remained unclear.

**Objective:**

To assess the role of environment, medical care and individual risks factors for *P*. *aeruginosa* colonization and infection.

**Study design and setting:**

A French multicentric prospective study involved ten ICUs for a five months period. Every adult patient newly hospitalized in ICUs with no *P*. *aeruginosa* carriage up to 48 hours after admission was included and weekly screened before discharge or death. Screening swabs were either rectal, sputum or oropharyngeal samples. Hydric environment was also sampled each week. Data on patient clinical features, environmental and device exposures, and antibiotics supports were regularly collected. Multivariate analysis was performed with a multistate model.

**Results:**

The overall prevalence of *P*. *aeruginosa* carriage was 15.3% (201/1314). Risk factors associated with patient colonization were: use of inactive antibiotics against *P*. *aeruginosa* (HR = 1.60 [1.15–2.21] p<0.01), tap water contamination at the entry in the room (HR = 1.66 [1.01–2.27] p<0.05) and mechanical invasive ventilation (HR = 4.70 [2.66–8.31] p<0.0001). Active antibiotics prevented from colonization (HR = 0.67 [0.48–0.93] p = 0.02) and from infection (HR = 0.64 [0.41–1.01] p = 0.05). Interaction between hydric environment antibiotics support was not statistically associated with patient colonization.

**Conclusion:**

Hydric contamination and antibiotics pressure seem to remain key independent risk factors in *P*. *aeruginosa* colonization. These results advocate the need to carry on preventive and targeted interventions toward healthcare associated infections.

## Introduction

Despite persistent and constant efforts made in Healthcare associated infections prevention, cross infections remain an issue especially in intensive care units (ICU). In 2012, 23% of patients acquired a healthcare associated infection during their hospitalization in an ICU that is a risk four times higher than in other hospital units [1].

Cross infections are associated with high mortality rates and higher costs by complicating patients’ cares and lengthening hospital stay [2–5].

Pathogens responsible for those infections are often multi-drug resistant (MDR) bacteria. Among those pathogens, Pseudomonas aeruginosa is often encountered [6] and is responsible for severe infections, difficult to manage, such as ventilator associated pneumonia, bacteremia or skin infections, mainly in immunocompromised patients [7] with already a poor baseline prognosis. Indeed this bacteria, once it has colonized the patient digestive mucosa or skin, can lead to infections by immune deficiency and skin or mucosa breach by indwelling invasive device for example. When focusing on the bacteria strains it seems that P.aeruginosa related infections are more likely to occur to P.aeruginosa colonized patients, which highlights the link between the two conditions [8–9].

*P*.*aeruginosa* transmission is known to be partly endogenous and exogenous but the precise roles of one and another remain uncertain. Human digestive flora has been described as the main source of endogenous transmission that can be enhanced by antibiotic pressure [8]. Hydric environment such as faucets, invasive devices exposure and other colonized patients are responsible for exogenous transmission. Medical staff has been identified as a key carrier from patients to patients (cross-transmission) [10]. Although genotyping methods allow tracking the spread of bacteria, the respective contribution of the various factors remains unclear.

Hence, the factors associated with *P*.*aeruginosa* colonization or infection are variable from one study to another [11] including: length of hospitalization [12] severity of underlying disease [12–14] exposure to invasive procedures[13–15], contamination of water tap [12,16–18], close contact with contaminated patients [14], antibiotic selective pressure [16–17]. The heterogeneity of the factors reported is due partly to the complexity of the measurement of exposures, the definition of the outcome and of the methods used for the analysis of the association with *P*.*aeruginosa* colonization or infection. In a previous report, we identified individual risk factors and environmental factors associated to *P*.*aeruginosa* acquisition in a cohort of 1314 patients hospitalized in ICU in France [19]. However, we did not distinguish between colonization and infection, both were merge in the same outcome although factors could be different in the two situations with different clinical consequences. The aim of our study was to assess the distinct role of environment, antibiotic and patient condition toward both colonization and infection, and to identify an interaction between environment and antibiotic selective pressure.

## Materials and methods

### Study setting

In 2009, an observational prospective multicentric study, entitled DYNAPYO, was performed, involving ten French ICUs for a five months study period. There were two surgical, four medical and four mixed ICUs (both medical and surgical units). Six university hospitals (Besançon, Bordeaux, Garches, Lyon, Montpellier and Paris) and two general hospitals (Lens and Tourcoing) took part in the study and included each one ICU, excepted for Besançon and Lyon which included two ICUs. These ICUs amounted between 9 and 20 beds and between 10 and 47 water taps. Data collection regarding environment and patient population was performed by trained healthcare professionals and was registered into a secure online electronic form.

### Sample size

In 2006 a pilot study was performed. In that study, the patients exposed to hydric contamination had a *P*.*aeruginosa* acquisition risk of 1.7. Within 6 months, the incidence of *P*.*aeruginosa* acquisition was 5.7%. To confirm such size effect with a statistical power of 80% and type I error of 5%, at least 112 events should be recorded. According to the expected incidence, it required to include around 2000 patients. However, the present study was stopped prematurely because the incidence of acquisition was higher than expected. Every newly hospitalized adult patient in ICUs with no *P*.*aeruginosa* carriage within the 48 first hours after admission was included. No *P*.*aeruginosa* carriage means that samples (rectal oropharyngeal or sputum swabs) were found negative at admission and within the 48 first hours. During the follow-up period, they were weekly screened (rectal oropharyngeal or sputum swabs) until they died or were discharged. Data were collected through medical record review: those about clinical and medical conditions and prior antibiotic use were collected at admission, and follow-up data such as hydric environment pressure, indwelling invasive device exposure and antibiotic support were regularly recorded.

To estimate the hydric environment pressure, faucets were weekly sampled in the morning before use and disinfection. Specific sample protocol has been described in the previous report [19]. No hygiene protocol modification could happen within the study period because of these results as the units were blinded.

Samples were then analyzed by the bacteriology laboratories where aerobic cultures were performed.

Data were made anonymous. All patients were informed of the survey and their rights. Signed consents were not required. Local ethical committee [Comité Consultatif sur le Traitement de l’Information en matière de Recherche dans le domaine de la Santé (CCTIRS) et Commission de l’Informatique et des Libertés (CNIL)] approved the study.

### Determination of colonization and infection

The first patient colonization and the first patient infection by *P*.*aeruginosa* were the two outcomes of interest. Patient colonization was defined as the presence of *P*.*aeruginosa* among at least one of the screening sample site from the 48^th^ hour until discharge. If any colonization was identified within the 48^th^ hour, patients were considered as imported cases and were not included in the analysis but still considered in exposure factor calculation. Colonization status was unknown for healthcare workers so that no change in usual isolation procedure occurred during the study.

Patient infection was defined according to current recommendations from REA-RAISIN network [20], as clinical and biological infection features associated with the isolation of the bacteria in any samples (blood culture, urine culture…).

### Main exposure factors

We focus on two exposure factors of main interest. First, hydric environment pressure was measured by sampling performed at the faucets located in the patient room: Tap water contamination at the entry in the patient room was the first main exposure factor. Antibiotic treatments prescribed since the day before, were the second main exposure factor and were daily recorded. We distinguished exclusive inactive antibiotics from active antibiotics against *P*.*aeruginosa* which includes at least one of the following: ureido and carboxypenicillins, antipseudomonal cephalosporins, carbapenems, colimycin fosfomycin, fluoroquinolones, and aminoglycosides. The antibiotic activity was determined by the theoretical sensivity of antibiotics on a wild-type *P*.*aeruginosa*.

### Other risk factors

Other individual risk factors were: age, sex, immucompromized status, IGSII score (i.e patient disease severity at the admission in ICUs predicting mortality), Charlson comorbidity index for chronic disease, cause of admission, prior antibiotics prescription before admission, prior hospitalization within the year prior to admission, history of prior carriage of *P*.*aeruginosa* (i.e prior colonization or diagnostic of *P*.*aeruginosa* infection). Environment risk factors were: any specialization of the ICU (medical, surgical or mixed), center and prior occupation by a colonized patient before entry in the room. Risk factors associated with medical care were: invasive mechanical ventilation exposure and broncho-endoscopy exposure.

### Statistical analysis

**Fig 1:**
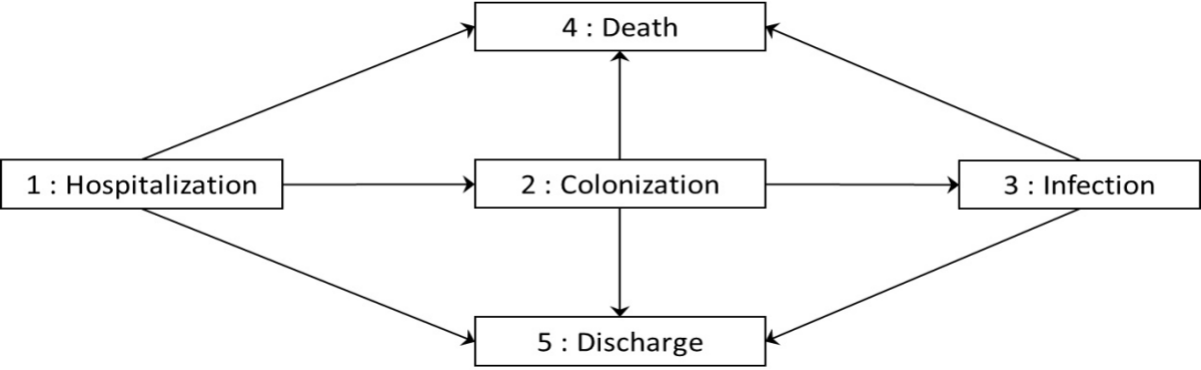
Multistate model representation.

Analysis was performed with a multi-state model that included five states and eight transitions (Fig 1). Baseline state 1—Hospitalization was the baseline status of patients hospitalized without colonization nor infection. States 2—Colonization and 3—Infection reflected the occurrence of the first episode of colonization and infection. We assumed that the state 3—Infection was systematically preceded by the state 2—Colonization. Arrows represent transitions from a state to another. The model assumed that transitions were markovian, which means that history of past states (i.e. duration in the state) was not taken into account in the estimation [21]. A discrete non-homogeneous model was assumed: discrete because time unit is the day; non-homogeneous because time-dependent variables were used as adjustment variables [22].

We estimated the associations between risk factors and events of interest by modelling transition intensities. The transition intensities represent the instantaneous risk of going from one state to another (e.g. acquiring infection from carrying *P*.*aeruginosa*).

Analyses were performed with the package “MSM” developed by C. Jackson in the R^®^ software [23]. Weekly screenings for both patients and water taps led to interval censoring which was taken into account by the model. Hydric environment exposure was assumed to be constant between two screenings. In case of punctual missing values regarding follow-up data, the package allows missing at random (MAR) imputation; in case of baseline or all along missing data, patients were removed from analyses. To minimize loss of patients, conditional imputation procedure was implemented to replace missing data for IGSII score, history of prior carriage of *P*.*aeruginosa*, prior hospitalization within the year prior to admission and prior antibiotics prescription before admission. To answer the aims of the study, we ran the model transition by transition with the following order of interest: “Hospitalization-Colonization”, “Colonization-Infection”, “Colonization-Death”, “Infection-Death” and “Hospitalization-Death”. We did not focus on the transition to discharge, as the event was only considered as a competing risk.

We first performed an univariable analysis including ten baseline factors and six binary time-dependent factors in each transition. Our three main exposure factors were forced in the model whatever their association, apart from hydric environment pressure which was specifically studied in the transition to colonization for the sake of clinical plausibility. We performed a multivariable analysis including significant variables with a p-value<25% using a step-by-step forward approach for each transition (p<5%). Adjustment for site was performed and we evaluated the interactions between hydric environment and both active and inactive antibiotics selective pressure in the final model in the transition leading to colonization. Hazard ratios and their 95% confidence intervals were provided as well as likelihood ratio test p-values. Log-linearity of the risk for quantitative variables has been checked.

## Results

### Study population

In the DYNAPYO cohort, 1700 patients were newly hospitalized, including 1314 *P*.*aeruginosa*-free patients who were enrolled in the study. Patients characteristics are shown in Tables 1 and 2.

**Table 1:** Patients characteristics at admission. Dynapyo cohort, France 2009.

Patient characteristics	Included patients (n = 1314)	
	Total	Proportion in %
**Sex**		
**Male**	772	58.8
**Female**	542	41.2
**Immunodeficiency**		
**Yes**	144	11.0
**No**	1160	88.2
**Unknown**	10	0.8
**Prior hospitalization in the year before admission**		
**Yes**	598	45.5
**No**	699	53.2
**Unknown**	17	1.3
**Prior carriage of *P*. *aeruginosa***		
**Yes**	27	2.1
**No**	1220	92.8
**Unknown**	67	5.1
**Surgery within 30 days before admission**		
**Yes**	283	21.5
**No**	1026	78.1
**Unknown**	5	0.4
**Prior antibiotic administration before admission**		
**Active antibiotics**	51	3.9
**Inactive antibiotics**	187	14.2
**Active and inactive antibiotics**	136	10.4
**No antibiotics**	866	65.9
**Unknown**	74	5.6
**Cause of hospitalization**		
**Coma**	388	29.5
**Acute respiratory insufficiency**	367	27.9
**Septic shock**	176	13.4
**Others**	383	29.2
**Intensive care unit (ICU)**		
**Medical ICU**	604	46.0
**Surgical ICU**	179	13.6
**Mixed ICU**	531	40.4
**Patient characteristics**	**Included patients (n = 1314)**	
	**Total**	**Median (IQR)**
**Age (in years)**	1314	59 (43–73)
**IGS-II score at admission**	1284	42 (30–56)
**CHARLSON index**	1314	1 (0–3)

**Table 2:** Exposure characteristics during the follow-up. Dynapyo cohort, France 2009.

Exposure characteristics	Included patients (n = 1314)	
	Total	Proportion in %
**Mechanical ventilation exposure >24h**		
**Yes**	903	68.7
**No**	407	31.0
**Unknown**	4	0.3
**Bronchoscopic endoscopy**		
**Yes**	127	9.7
**No**	1183	90.0
**Unknown**	4	0.3
**Exposure to a room previously occupied by a colonized patient**		
**Yes**	320	24.4
**No**	990	75.3
**Unknown**	4	0.3
**Tap water of the room contamined by *P*.*aeruginosa***		
**Yes**	315	24.0
**No**	995	75.7
**Unknown**	4	0.3
**Exposure to active antibiotics**		
**Yes**	491	37.4
**No**	818	62.2
**Unknown**	5	0.4
**Exposure to inactive antibiotics**		
**Yes**	601	45.7
**No**	708	53.9
**Unknown**	5	0.4

The median age was 59 years and 11% of patients were immunocompromised. The median IGSII score was 42 points (Interquartile Range IQR: 30; 56). The median (IQR) length of stay was 6 days (3; 13) and 232 patients (17.7%) died in a median delay of 7.5 days (3; 15). First colonization and first infection were respectively estimated to occur at 10 days (7; 16) and 12 days (8; 19). During the follow-up, 70% were mechanically ventilated and among them, almost 90% were ventilated at the admission.

A total of 855 positive hydric environment samples among 4999 screened were positive for *P*. *aeruginosa* (17.1%).

### Colonization and infection incidences

Among the 1314 patients, 201 patients were colonized during a total of 11 915 days follow-up, corresponding to an incidence of 16.9 colonizations per 1000 patient-days (_95%_CI [14.6–19.2]). An infection was observed in 87 patients during a 12 608 days of follow-up, representing 43.3% of *P*.*aeruginosa* carriers and an incidence of 6.9 infections per 1000 patient-days (_95%_ CI [5.5–8.3]).

### Risk factors analysis

**Fig 2:**
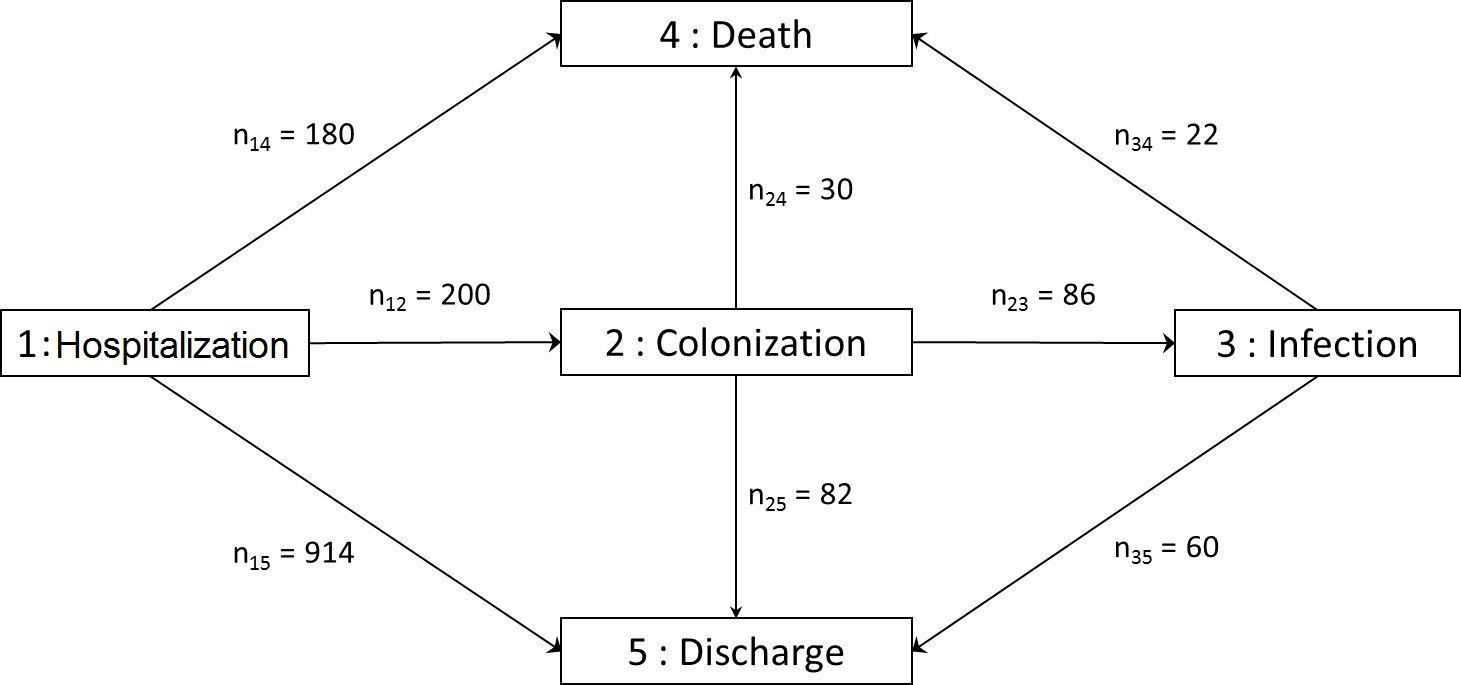
Multistate model and number of subjects switching from one state to another. By definition, the 1305 patients started their hospitalization in state 1–hospitalization. n_ij_: number of patients moving from state i to state j.

1305 patients were included in the analysis in the state 1–Hospitalization whereas 9 patients were excluded from this analysis because of missing data for one or more variables. Among the 1305 patients, 180+200+914 moved to another state, meaning that 11 stayed in the state 1–Hospitalization at the end of the study (right-censoring). Also two and four patients stayed respectively in the states 2–Colonization and 3–Infection at the end of the study and were right-censored. Fig 2 shows the evolution of those 1305 patients during hospitalization.

Table 3 shows the main results after adjustment for the site.

**Table 3:** Multistate model after adjustment for the site: Risk factors for *P*. *aeruginosa* colonization, infection and risk factors for death. DYNAPYO Cohort–2009.

**Variables**	**Hospitalization–Colonization**			**Colonization–Infection**					
	**(n**_**1**_ **= 1305) (n**_**2**_ **= 200)**			**(n**_**2**_ **= 200) (n**_**3**_ **= 86)**					
	**HR**	_**95%**_**CI**	**p-value**	**HR**	_**95%**_**CI**	**p-value**			
**Contaminated hydric environment exposure at the entry room**	1.66	1.01–2.75	<0.05						
**Active antibiotics exposure**	0.67	0.48–0.93	0.02	0.64	0.41–1.01	<0.05			
**Inactive antibiotics exposure**	1.60	1.15–2.21	<0.01	0.77	0.47–1.26	0.30			
**Prior *P*.*aeruginosa* carriage**	4.03	1.96–8.27	<0.01						
**Invasive mechanical ventilation**	4.70	2.66–8.31	<0.0001						
**Variables**	**Hospitalization–Death**			**Colonization–Death**			**Infection–Death**		
	**(n**_**1**_ **= 1305) (n**_**4**_ **= 180)**			**(n**_**2**_ **= 200) (n**_**4**_ **= 30)**			**(n**_**3**_ **= 86) (n**_**4**_ **= 22)**		
	**HR**	_**95%**_**CI**	**p-value**	**HR**	_**95%**_**CI**	**p-value**	**HR**	_**95%**_**CI**	**p-value**
**Active antibiotics exposure**	0.65	0.47–0.90	<0.01	0.41	0.19–0.87	0.02	1.67	0.35–8.07	0.51
**Inactive antibiotics exposure**	0.83	0.60–1.14	0.24	0.86	0.36–2.04	0.77	0.66	0.25–1.71	0.41
**Age (↗ 10 years)**	1.12	1.01–1.23	0.03						
**IGS II score up to 24 hours (↗ 10 points)**	1.46	1.34–1.60	<0.0001	1.33	1.06–1.66	0.01			
**Admission diagnosis (ref.Others)**			0.06			0.02			
**Coma**	1.79	1.13–2.84		3.69	1.20–11.33				
**Acute respiratory distress**	1.21	0.75–1.96		2.06	0.66–6.41				
**Septic shock**	1.16	0.70–1.92		0.51	0.10–2.68				

HR = Hazard ratio

_95%_ CI = 95% Confidence interval

p-value: obtained with Likehood ratio test

Interactions:

Contaminated hydric environment *Active antibiotics exposure p = 0.78

Contaminated hydric environment *Inactive antibiotics exposure p = 0.28

The risk of colonization was significantly increased when inactive antibiotic were prescribed (HR = 1.60; _95%_CI = [1.15–2.21]), whereas it was decreased when active antibiotic were prescribed (HR = 0.67; _95%_CI = [0.48–0.93]). Tap water contamination at the entry in the patient room enhanced the risk of colonization by +66% (HR = 1.66; _95%_CI = [1.01–2.75]). History of *P*.*aeruginosa* carriage and invasive ventilation were also significant independent risk factors of *P*.*aeruginosa* colonization: HR = 4.03; _95%_CI = [1.96–8.27] and HR = 4.70; _95%_CI = [2.66–8.31], respectively. Tests for interactions between the hydric environment and inactive or active antibiotics were not statistically significant (p-values 0.28 and 0.78, respectively).

The factors associated with *P*.*aeruginosa* infection were limited. Among colonized patients, the prescription of active antibiotic tended to be associated with a lower risk of infection occurrence (HR = 0.64; _95%_CI = [0.41–1.01]). There was no significant association with inactive antibiotic support (HR = 0.77; _95%_CI = [0.47–1.26]).

Older age (HR = 1.12; _95%_CI = [1.01–1.23]) increased the risk of death during hospitalization. Whatever the colonized status, a higher IGSII score (HR = 1.46; _95%_CI = [1.34–1.60] and HR = 1.33; _95%_CI = [1.06–1.66]) and the diagnosis of coma (HR = 1.79; _95%_CI = [1.13–2.84] and HR = 3.69; _95%_CI = [1.20–11.33]) were associated with an increased risk of death.

## Discussion

In an endemic situation, this study showed the constant presence and role played by *P aeruginosa* with incidence density rates of 16.9 colonizations and 6.9 infections per 1000 patient-days. Hydric environment was identified as a consistent and independent risk factor, but also individual and health risk factors such as prior carriage of the bacteria, mechanical ventilation and antibiotic use were found to contribute to the transmission of the bacteria. No interaction between antibiotic use and hydric environment contamination was detected. Therefore, an inappropriate prescription of antibiotics does not seem to increase the risk of contamination by the hydric environment.

These results highlighted the consistent role of the hydric environment in *P*.*aeruginosa* transmission and the independent effect of both active and inactive antibiotics support in colonization occurrence. The prevalence of colonized tap water was of 17.1%, quite similarly in others studies [24]. Our results on risk factors are consistent with those from previously published studies [13,16,18,25,26]. Environment has been identified as a major risk factor in the exogenous transmission. Petignat et al. have shown the impact of infection control measures targeting faucets toward *P*.*aeruginosa* colonization [27]. Prior carriage of *P*.*aeruginosa* and mechanical ventilation were also found as important risk factors [11]. Active antibiotics are described as protective factors [14,17,28], whereas inactive antibiotics have already been identified as risk factors [8,16]. In the present study, the antibiotics activity was defined according to the theoretical knowledge of the effect of each molecule and not by the antibiogram for any participant. This could represent an important limit in our study. However, the observed opposite effects of active and inactive antibiotics is expected: inactive antibiotics, involving an unbalance of the digestive microflora can indeed allow the bacteria to proliferate, in contrary to active antibiotics which remove the bacteria.

Although we did not find any evidence for an interaction between the environment and antibiotics, it does not mean that there was none. Either there is no interactive role between antibiotics and environment, or there is one which could have been missed by a lack of statistical power or by an ignoring confounding factor.

Routine screening for *P*.*aeruginosa* carriage is not recommended [29], but this could be questionned: first we identified prior *P*.*aeruginosa* carriage as a risk factor which could be linked to clinical predisposition to acquire the bacteria once again. Second, we found that 43% of colonized patients developed infectious conditions, and these results are consistent with Gomez-Zorilla et al. [8] who ascertained that infections occurred much frequently among colonized than non-colonized patients (39% vs 3.4% p<0.001). Third, we highlighted the protective role of active antibiotics and featured that deaths occurred more likely with infected patients (22/86) than colonized patients (30/200). Thus, according to medical history and condition, for example for immunocompromised patients or among those that received common antibiotics (which are mainly inefficient against the bacteria), this screening associated with genotyping resistance test, could avoid delayed efficient antibiotics and so, avoid death [30].

Thanks to the use of multistate model that allows to distinguish colonization from infection, we could demonstrate that indeed very few factors were associated with the risk of infection: the exposure to active antibiotic especially. Also, usual risk factors of death were isolated (age, IGSII, coma).

Furthermore as we carefully checked the link between anterior contaminated samples in faucets and patient incident colonization, these results strengthen what we found in the risk factors analysis.

Although a seven days interval screening seems to be a good compromise between feasibility and costs [24,26], test performances with a moderate sensitivity [31] and number of screening samples could have led to classification error. Colonization incidence could have been overestimated because of wrong inclusion of unknown *P*.*aeruginosa* carriers who had been then identified as incident cases. Hence this could explain the association between prior carriage of *P*.*aeruginosa* and colonization. Also, water tap sampling was also weekly collected and previous notes can also be applied: water tap status was considered constant until the next screening. This situation has probably led to a decrease of the association between water tap pressure and colonization or infection. However, a more intense screening is difficult to organize. Future studies may take advantage of new technologies such as the i-BIRD project [10] in which spatio-temporal data of contact between healthcare workers are measured by sensitive sensors.

In conclusion, hydric contamination and antibiotics pressure seem to remain key independent risk factors in *P*.*aeruginosa* colonization. These results advocate the need to carry on preventive and targeted interventions toward healthcare associated infections. This study encourages the settlement of cautious healthcare associated infections prevention measures such as hands hygiene, respect of aseptic rules and more regular faucets maintenance.
